# Prevalence and Clinical Characteristics of Alopecia Areata at a Tertiary Care Center in Saudi Arabia

**DOI:** 10.1155/2020/7194270

**Published:** 2020-03-12

**Authors:** Aysha A. Alshahrani, Rawan Al-Tuwaijri, Zainah A. Abuoliat, Mesnad Alyabsi, Mohammed I. AlJasser, Rayan Alkhodair

**Affiliations:** ^1^College of Medicine, King Saud Bin Abdulaziz University for Health Sciences, Riyadh, Saudi Arabia; ^2^Division of Family Medicine, King Fahad Medical City, Riyadh, Saudi Arabia; ^3^Division of Dermatology, Ministry of National Guard Health Affairs, Riyadh, Saudi Arabia; ^4^King Abdullah International Medical Research Center, Riyadh, Saudi Arabia

## Abstract

**Introduction:**

Alopecia areata (AA) is a common autoimmune disorder of hair follicles characterized by patches on nonscarring hair loss. Reports of prevalence and clinical characteristic of AA in Saudi Arabia are limited. The aim of our study is to describe the prevalence and clinical characteristics of Saudi patients with AA.

**Materials and Methods:**

A retrospective cross-sectional study was conducted at King Abdulaziz Medical City in Riyadh, Saudi Arabia. All patients diagnosed with AA between January 2016 and December 2017 were included. Data included patient demographics, type of AA, disease duration, family history of AA, and comorbid autoimmune diseases.

**Results:**

A total of 216 patients with AA were included. The overall prevalence of AA was approximately 2.3%. The mean disease duration at the time of presentation was 2 months while the mean age of onset was 25.61 years. The most common type of AA in both adult and pediatric groups was the patchy type involving the scalp. Comorbid diseases were found in 32.41% of patients. Common associated conditions included hypothyroidism, diabetes mellitus, and atopic diseases.

**Conclusion:**

The overall prevalence of AA among a population of Saudi patients is 2.3%. AA prevalence is higher in pediatrics than adults. Common comorbid conditions include hypothyroidism, diabetes mellitus, and atopic diseases.

## 1. Introduction

Alopecia areata (AA) is a nonscarring alopecia caused by a T-cell-mediated autoimmune destruction of hair follicles [1]. The general population has an approximately 2% risk to develop AA at any time of their life [2, 3]. The worldwide incidence of AA varies from 2.1%, 0.7%, to 3.8% in USA, India, and Singapore, respectively [4]. Reports showed that there is no gender predominance in AA (5). AA is considered as a disease of all age group; however, most patients present at age of 21–40 years [5]. Globally, AA onset was at 25 and 36 years of age in Singapore and USA, respectively [4].

There are several hypotheses regarding AA pathogenesis. It has been suggested that viral and bacterial infection, endocrine, autoimmune, psychological, and genetic factors may play a role in AA pathogenesis [1]. One of the most important risk factors of developing AA is family history which is portrayed in one of the studies that showed the lifetime risk of AA was 7.1%, 7.8%, and 5.7% in siblings, parents, and children of patients with AA, respectively [5, 6]. Another risk factor is atopy as shown in 60% of adults and 25% of pediatric patients with AA who either had personal or family history of atopy [5, 7, 8].

AA has three main variants which are patchy AA (localized hairless areas), alopecia totalis (entire scalp affected), and alopecia universalis affecting all body surface area [9]. Other AA subtypes include ophiasis (band-like alopecia in the occipital and temporal scalp), sisaipho (central hair loss sparing the marginal hair line), and diffuse form [5]. AA severity can be divided into mild (≤3 patches), moderate (≥3 patches without alopecia totalis or universalis), and severe (alopecia totalis, universalis, and ophiasis) [10].

There are different treatment modalities for AA patients starting from minimal approach with topical or injectable corticosteroids to more extensive one with systemic immunomodulators. Treatment response varies depending on the severity of AA, age of onset, and other factors [11, 12]. Despite AA being commonly seen in daily practice among Saudi patients, studies are limited and outdated in our population [13]. This study aims to describe the prevalence and clinical characteristics of patients diagnosed with AA at King Abdulaziz Medical City in Riyadh, Saudi Arabia.

## 2. Methods

This study was a retrospective cross-sectional study conducted at King Abdulaziz Medical city. It included all patients diagnosed with AA between January 2016 and December 2017. Data were retrospectively collected by reviewing the electronic medical records. Data included patient demographics, type of AA, disease duration, family history of AA, and comorbid autoimmune diseases. Severity of AA was divided into patchy hairless area as mild/moderate and alopecia totalis, universalis, or ophiasis as severe. Sample size calculation was performed using Raosoft.com with a 5% margin of error and 95% confidence level. The minimum recommended sample size was 197. A convenience sampling technique was used. Descriptive statistics were presented as frequencies and percentages for categorical variables (age categories, gender, types of AA, family history, presence of comorbidities, and autoimmune diseases). Mean ± standard deviation was used for numerical variables (age at onset and disease duration). The significance of associated comorbidities and autoimmune diseases in alopecia areata patients was presented as a 95% confidence interval. A *P* value <0.05 was considered as statistically significant. Data were analyzed using SAS statistical software version 9.4 (SAS Institute Inc. Cary, NC). This study was approved by the Institutional Review Board in King Abdullah International Medical Research Center (study number RC18/050/R).

## 3. Results

A total of 216 patients with AA were included (Table 1). During the study period, 9,317 new patients were referred to the dermatology clinic. Therefore, the overall prevalence of AA was estimated to be approximately 2.3%. Pediatric and adult cases accounted for approximately 4.24% and 2% of all new referrals to the pediatric and adult dermatology clinics, respectively. The mean age at onset was 25.61 ± 12.92 years. A disease onset before the age of 15 years was observed in 22.6% of cases. The mean disease duration at the time of presentation was 2 months. Males with AA were more than females with a ratio of 1.37 : 1. A family history of AA was positive in 6% of patients. The most common type of AA in both adult and pediatric groups was the patchy type involving the scalp (Table 2). Most of the patients had mild-to-moderate AA 187 (86.6%) while the rest had a severe AA 29 (13.4%). Children were more likely to have severe AA as compared to adults (*P* < 0.01). There was no statistically significant association between the severity of AA and gender or comorbidities (Table 3).

Associated diseases were found in 70 (32.41%) patients (Table 4). Hypothyroidism and diabetes mellitus were among the most common comorbidities. Atopy was found in 13.89% of patients with asthma being the most common followed by atopic dermatitis and allergic rhinitis. A positive family history of autoimmune disease was found in 5.5% of cases. Response to therapy was noted in 46.3% of patients, and spontaneous hair regrowth was found in 8.8%.

## 4. Discussion

Although AA is stressful disorder with social stigma, studies on its prevalence and clinical characteristics are limited in Saudi Arabia. The prevalence of AA in our study was 2.3% which is comparable with the worldwide figures [14]. In the United States, the prevalence of AA was approximately 2% [15] while it is slightly more in Japan reaching 2.45% [16]. Although AA is a disease of all ages, it is more prevalent in the younger age group. In this study, the prevalence in children was 4.24% with lower prevalence in adults (2%). In one meta-analysis, similar findings were demonstrated as higher prevalence of AA in children 1.83% (1.21–2.58%) compared to adults 1.64% (1.30–2.03%) [17].

Previous studies showed that patients with disease onset in the first 2 decades of life had more severe AA [7, 8]. In our study, there was a statistically significant difference in terms of severity between adults and children. Pediatric patients were more likely to have severe AA as compared with adults (*P* < 0.01).

The mean age of onset of AA in the present study was 25.61. This is similar to some previous studies of Asians with AA. The reported age of onset was 25.2, 28.98, 36.3, and 32.2 in Singapore, China, USA, and Taiwan, respectively [4]. Studies in the literature with regard to gender predominance in AA are conflicting. A systematic review concluded that there is no difference in the incidence of AA between males and females [4]. Our study showed a male predominance of AA while other studies showed female predominance [18]. AA has different types with patchy scalp being the most predominant type in our study as shown in agreement with previous studies [4].

The association of AA with other autoimmune disease had variable results in the literature. Some studies showed that AA is not related to other autoimmune diseases [19, 20]. However, many other studies demonstrated that AA is associated with several autoimmune diseases [21–23]. In our study, the 3 most commonly reported comorbidities were hypothyroidism, asthma, and diabetes mellitus. A previous local study showed an association between AA and thyroid disease [21]. Atopic diseases were common in our patients, which is in agreement with the findings of Al-Khawajah's study [13].

Our study has several limitations. We have conducted a retrospective chart review as the main source for data collection. Another limitation is the relatively small sample size.

## 5. Conclusion

The overall prevalence of AA in our study population is 2.3%. AA prevalence is higher among children than adults. Furthermore, children were more likely to have severe AA. The severity of AA was not associated with gender or the presence of comorbidities. Several comorbid conditions were found to be common in our patients including hypothyroidism, diabetes mellitus, and atopic diseases. Future prospective studies with larger sample size might be required to further characterize clinical features, risk factors, and treatment response among Saudi patients with AA.

## Figures and Tables

**Table 1:** Patient characteristics (*N* = 216).

Characteristic	*N*	%
Mean age at onset (SD)	25.61 (12.92)	
Gender		
Male	125	57.87
Female	91	42.13
Adult (≥14 years old)	167	77.3
Pediatrics (<14 years old)	49	22.69
Family history of alopecia areata	13	6
Family history of autoimmune diseases	12	5.5
Disease status		
(i) Respond to treatment	100	46.30
(ii) Active	18	8.33
(iii) Stable	18	8.33
(iv) Unknown	61	28.24
(v) Hair regrowth without treatment	19	8.8

**Table 2:** The frequency of alopecia areata types.

Types of alopecia areata	Adults *n* (%)	Pediatrics *n* (%)	Overall *n* (%)
Patchy scalp	83 (49.7)	22 (44.89)	105 (48.6)
Patchy scalp + patchy beard	14 (8.38)	0	14 (6.48)
Patchy scalp + patchy limbs	1 (0.6)	0	1 (0.46)
Patchy scalp + patchy eyebrows	1 (0.6)	4 (8.16)	5 (2.3)
Patchy scalp + patchy eyelashes + patchy eyebrows	1 (0.6)	4 (8.16)	5 (2.3)
Patchy scalp + patchy beard + patchy eyebrows	1 (0.6)	0	1 (0.46)
Patchy eyebrows	3 (1.8)	5 (10.2)	8 (3.7)
Patchy limbs	2 (1.2)	0	2 (0.92)
Patchy beard	46 (27.5)	0	46 (21.3)
Totalis\subtotalis	7 (4.19)	3 (6.12)	10 (4.63)
Universalis	1 (0.6)	3 (6.12)	4 (1.85)
Diffuse	2 (1.2)	1 (2.04)	3 (1.39)
Ophiasis	5 (2.99)	7 (14.29)	12 (5.56)
Total	167 (77.31)	49 (22.69)	216 (100)

**Table 3:** Factors associated with severity in alopecia areata patients.

	Mild-to-moderate (*n* = 187)	Severe (*n* = 29)	*P* value
Gender			
Male (*n* = 125)	112 (89.6%)	13 (10.4%)	0.12
Female (*n* = 91)	75 (82.4%)	16 (17.6%)	
Age			
Adult (*n* = 167)	152 (89.4%)	15 (10.6%)	<0.01
Pediatric (*n* = 49)	35 (71.4%)	14 (28.6%)	
Comorbidity			
Yes (*n* = 70)	58 (82.9%)	12 (17.1%)	0.26
No (*n* = 146)	129 (88.4%)	17 (11.6%)	

**Table 4:** The association of comorbidities with severity in alopecia areata patients.

Comorbidity	Mild-to-moderate (*n* = 187)	Severe (*n* = 29)	Total *n* (%)
Hypothyroidism	15	3	18 (8.33)
Asthma	14	2	16 (7.41)
Diabetes mellitus	9	2	11 (5.09)
Atopic dermatitis	5	3	8 (3.70)
Allergic rhinitis	6	0	6 (2.78)
Psoriasis	3	1	4 (1.85)
Vitiligo	3	0	3 (1.39)
Rheumatoid arthritis	1	1	2 (0.93)
Systemic lupus erythematous	1	0	1 (0.46)
Seborrheic dermatitis	1	0	1 (0.46)
Total	58	12	70 (32.41)

## Data Availability

The data used to support the findings of this study are included within the article.
